# Uses of coherent Raman scattering microscopy in neuroscience

**DOI:** 10.3389/fnins.2025.1715954

**Published:** 2026-01-29

**Authors:** Mutsuo Nuriya

**Affiliations:** 1 Department of Pharmacology, Keio University School of Medicine, Tokyo, Japan; 2 Graduate School of Environment and Information Sciences, Yokohama National University, Yokohama, Kanagawa, Japan

**Keywords:** chemical imaging, coherent Raman scattering, multimodal imaging, multiphoton microscopy, Raman imaging, Raman tag

## Abstract

Multiphoton microscopy allows the imaging of biological phenomena deep within brain tissues and has greatly advanced knowledge in neuroscience. However, many optical phenomena other than the multiphoton excitation of fluorophores in nonlinear optics are underrecognized. Coherent Raman scattering (CRS) uses multiple photons to boost weak Raman scattering. CRS has been used to enable molecular vibration-dependent contrast imaging of tissues and has been particularly useful for pathophysiological investigations of brain tissues. Recently, the combination of CRS with Raman-active bio-orthogonal chemical bonds or groups has proven particularly powerful for visualizing molecules not detectable by fluorescence imaging. This review introduces a new and exciting imaging strategy and its applications in neuroscience.

## 1 Introduction

**Figure 1:**
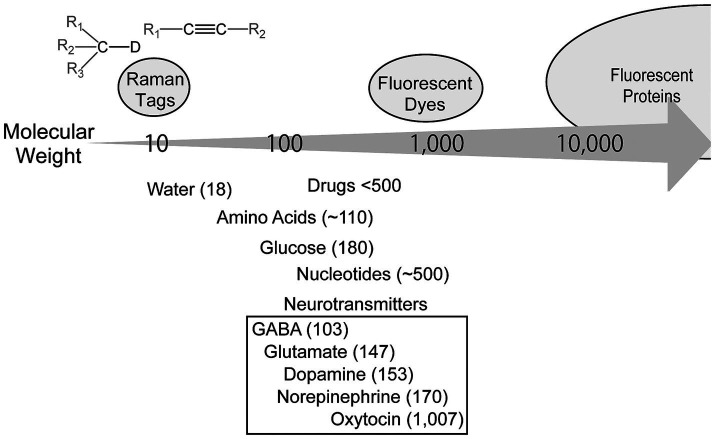
Molecular weights of the imaging tags and target bioactive molecules. The molecular weights of the imaging tags and selected bioactive molecules related to neuroscience are also shown.

Technological breakthroughs have led to significant progress in neuroscience. The introduction of multiphoton microscopy is arguably a key technical breakthrough in neuroscience. Using highly tissue-penetrating near-infrared light and the simultaneous interactions of multiple photons with molecules that only occur at highly photon-dense spatially restricted focal points, three-dimensional high-spatial-resolution imaging is possible in deep brain tissue. Moreover, because photochemical reactions are restricted to the focal volume, unintended and unfavorable reactions, including photodamage and photobleaching, are significantly reduced, enabling low-toxicity long-term imaging. Together with intensive efforts to develop various fluorescent dyes/proteins, two-photon excitation fluorescence microscopy has emerged as a pivotal tool for investigating the physiology, pathophysiology, and pharmacology of the brain, and has brought unprecedented knowledge to neuroscience. However, large fluorophores with molecular weights of several hundred for organic dyes, and even larger for fluorescent proteins (Figure 1), impose critical limitations. The addition of large fluorophores alters the physical, chemical, and biological properties of small molecules; therefore, fluorescently tagged molecules no longer function as reliable probes for the original molecules. Many bioactive molecules related to neuroscience are small. The molecular weight of most neurotransmitters is <300, and most drugs have a molecular weight of <500 (Figure 1). Fluorescence tagging cannot be applied for these important molecules; therefore, their dynamics cannot be directly imaged. Consequently, optical imaging that does not rely on fluorescence is required to overcome these limitations. Coherent Raman scattering (CRS) is a promising optical modality for resolving this issue and has shown great potential to empower life science research through its 25 years of history (Cheng et al., 2025; Min et al., 2025; Xie, 2025). This review introduces the applications of CRS microscopy in neuroscience with a special focus on Raman tag imaging.

## 2 CRS microscopy and Raman tags

**Figure 2:**
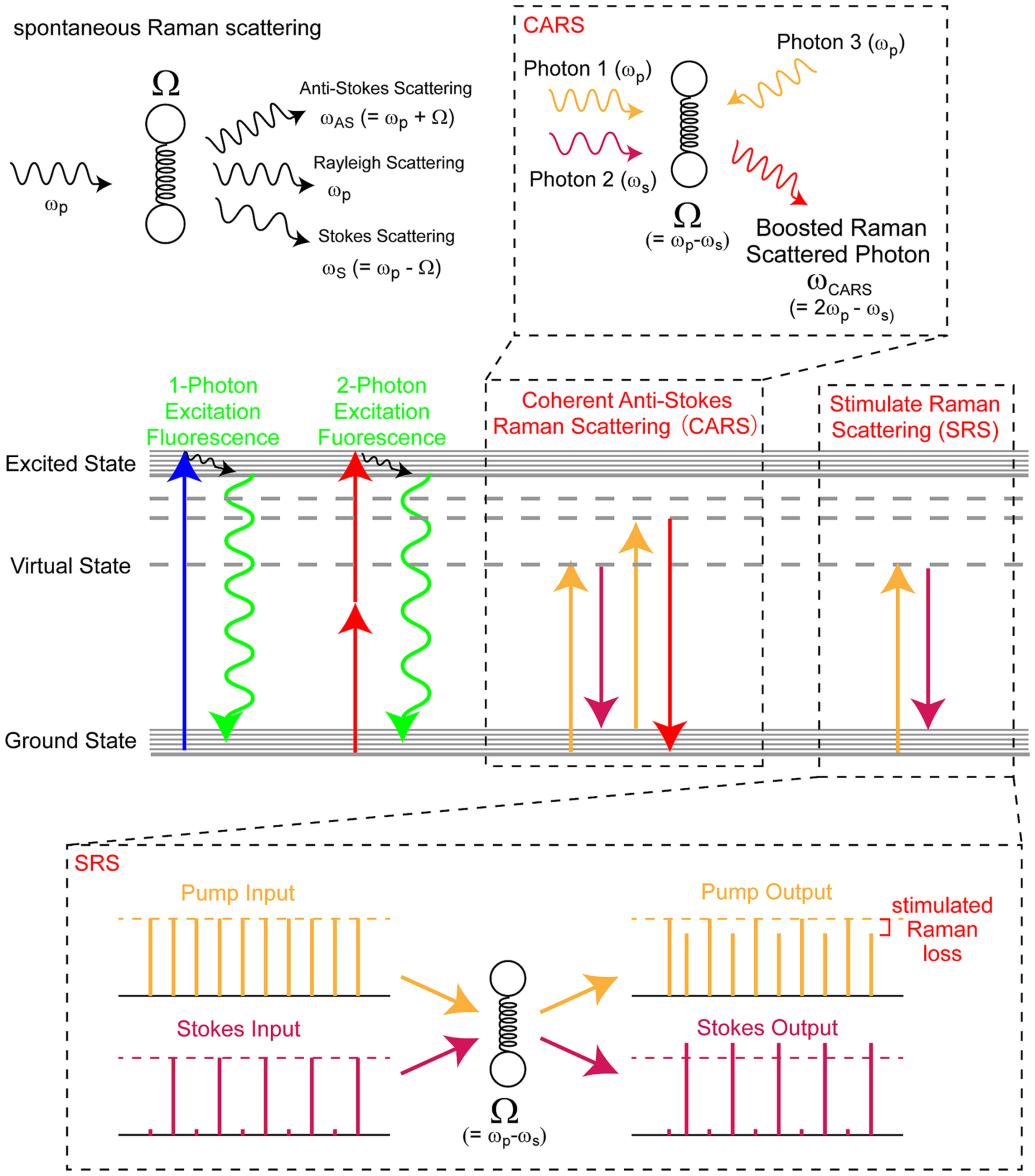
Schematic diagram of Raman scattering. The incident light (frequency *ω*) interacts with a molecule with frequency *Ω* to generate Rayleigh scattering (ω), anti-Stokes scattering (ω + Ω), and Stokes scattering (ω − Ω) light (top left). CARS and SRS are similar to two-photon excitation fluorescence in that they utilize multiple photons; however, instead of exciting the fluorophores, they resonantly enhance Raman scattering (middle). For both CARS and SRS, pump (frequency: ω_p_) and Stokes (ω_S_) beams are tuned so that the difference in their frequencies corresponds to the frequency of the target vibration (Ω = ω_p_ − ω_S_). In the CARS imaging, the newly generated anti-Stokes photons are collected (top right), whereas the stimulated Raman loss of the pump beam by a periodically modulated Stokes beam is detected in the SRS (bottom). CARS, coherent anti-Stokes Raman scattering; SRS, stimulated Raman scattering.

Raman scattering microscopy visualizes targets based on the vibrational energies of molecules and has long been explored for use in biology (Figure 2). Although this is a powerful tool for imaging tissues in a label-free manner, its application in neuroscience is hampered by several critical limitations. Among them are the low efficacy of Raman scattering, low tissue penetration, and difficulty in the specific imaging of target molecules. The introduction of CRS and Raman tags can resolve these issues.

In CRS microscopy, two photons of different wavelengths are introduced into the observation samples. When the difference in the energy of these photons matches the vibrational energy of the target molecules, the vibration is resonantly excited, and the efficacy of the Raman scattering of the corresponding molecular vibration is significantly enhanced (Figure 2). Stimulated Raman scattering (SRS) and coherent anti-Stokes Raman scattering (CARS) are the two major CRS microscopy techniques currently used (Zumbusch et al., 1999; Freudiger et al., 2008; Saar et al., 2010). Importantly, because CRS is achieved through the interaction of molecules with multiple photons in the near-infrared region, it has the benefits of multiphoton excitation fluorescence microscopy. Furthermore, the laser sources needed for CRS can also satisfy the requirements for the multiphoton excitation of fluorophores, realizing the simultaneous acquisition of Raman scattering images and fluorescence images of dyes/proteins. Because Raman and fluorescence can provide complementary information, these properties are ideal for delineating complex phenomena inside the fragile and highly scattering brain tissue.

Although CRS overcomes the sensitivity and tissue penetration issues, the limitations of specific imaging remain. This is because Raman scattering is based on the vibrational energy of chemical bonds and not on the overall identity of molecules; therefore, molecules that share similar chemical bonds cannot be distinguished. Because cells synthesize various molecules from a limited number of original compounds, most molecules existing in cells and tissues share chemical bonds. Therefore, specific Raman imaging of target molecules can rarely be achieved using label-free imaging. Raman tags, a groundbreaking technology, were introduced to resolve this issue. This concept is similar to that of a fluorescent tag; however, the tag is a Raman-active chemical group rather than a fluorophore. Raman tags include deuterium-containing bonds (e.g., —C—D) and alkynyl group (—C ≡ C—) (Qian et al., 2025), which do not exist in biological samples. Importantly, these Raman tags have uniquely identifiable Raman scattering signals in a so called “silent region” of the Raman spectrum, defined as the spectral region where biological materials have only low Raman scattering signals. The biggest difference between these two modalities lies in their sizes: fluorophores have large molecular weights, normally several hundred, whereas Raman-active chemical bonds can be as small as one for the deuterium tag (Figure 1). Therefore, important bioactive molecules that are inadequate to be tagged with fluorophores can be tagged and potentially visualized by Raman imaging.

This concept was first introduced in life sciences as a specific imaging technique for nucleotides (Yamakoshi et al., 2011). This strategy has been extended, and various types of alkyne-tagged small bioactive molecules have been synthesized and used to monitor their dynamics in living cells (Wei et al., 2014). Alkyne-based Raman tags have expanded significantly through the introduction of different isotopes and functional groups, thereby increasing the number of distinct Raman tags available for Raman imaging (Wei et al., 2017; Hu et al., 2018; Qian and Min, 2022). This series of studies demonstrated another potential advantage of Raman-tag imaging, namely, its multicolor capacity. Because fluorescent dyes/proteins have broad emission spectra, the number of fluorophores that can be imaged simultaneously is limited owing to spectral overlap. Although different strategies have been developed to increase this number, conventional fluorescence imaging is limited to three or four colors. For Raman tag imaging, particularly for alkyne-based probes, the Raman spectra are very narrow. Therefore, the number of Raman tags simultaneously imaged without significant spectral mixing was notably higher than that of fluorescent imaging, thus realizing more than 10-color simultaneous imaging (Wei et al., 2017; Hu et al., 2018).

Although considerably smaller than fluorophores, alkyne tags still have a molecular weight of >20, which may alter the physicochemical and biological properties of the target molecules. In this respect, deuterium tags (e.g., C—D bonds) are even smaller and therefore have a smaller risk of perturbation. An additional advantage of deuterium tags is that many deuterated biological compounds have already been developed and have become available, unlike alkyne-tagged molecules that need to be synthesized *de novo*. A disadvantage of this tag is that the Raman signal of the individual C—D bonds is weaker than that of alkyne-based Raman tags (Yamakoshi et al., 2012), necessitating the introduction of multiple substitutions and/or higher concentrations of the target molecules for detection.

## 3 Applications in neuroscience

### 3.1 Metabolic imaging

Cellular activities are supported by the dynamics of macromolecules, which are composed of small building blocks such as amino acids with a mean molecular weight of ~110. Traditionally, the metabolic activity has been tracked using radioisotopes. Although powerful, they lack spatial and temporal resolution, and cannot be performed in living cells. These limitations can be overcome by imaging Raman-tagged molecules using CRS microscopes (Wei et al., 2014). Shortly after the original study, this technique was applied to neurons. Physiological metabolic activities and changes under pathological conditions were monitored by feeding Raman-tagged small building blocks of macromolecules to organotypic hippocampal slice cultures (Hu et al., 2016). The introduction of different Raman-tagged building blocks of macromolecules allows monitoring of the metabolic activities of DNA, RNA, proteins, and lipids. Later, the strength of CRS imaging over conventional isotope labeling was demonstrated by utilizing the rich information buried in Raman spectra to achieve versatile and comprehensive metabolic imaging; spectral information was used to distinguish different types of metabolites. Animals were fed deuterated water (D_2_O), and carbon–deuterium (C—D) bonds generated by the metabolic incorporation of D_2_O were monitored by spectral unmixing to distinguish the metabolites (Shi et al., 2018).

As the brain is known for its high demand for glucose, a detailed understanding of glucose metabolism is essential for understanding its physiology and pathophysiology. Raman tagging and CRS also enable imaging of this small molecule; glucose uptake can be imaged using an alkyne-tagged analog of glucose in neurons and brain slices (Hu et al., 2015). Once incorporated into the cells, glucose is metabolized and functions as a source of bioactive compounds. Again, these metabolic activities have been traced in animals by spectral tracing of deuterated glucose included in feed (Zhang et al., 2019). Taken together, combinations of Raman-tagged probes, spectral unmixing and other tools together with simultaneous fluorescence imaging of cellular markers can delineate intricate metabolic network activities of neurons and glial cells in brain tissues.

### 3.2 Neurotransmitter imaging

Tools for imaging neurotransmission are extremely valuable for understanding the chemical transmission between neurons and astrocytes. However, this has been a big challenge, as neurotransmitters are small; for example, the molecular weights of glutamate, GABA, and dopamine are 147, 103, and 153, respectively, making the fluorescence-tagging approach unapplicable. Tremendous efforts have been made to visualize neurotransmission by developing fluorescent sensors for neurotransmitters, typically by conjugating fluorescent proteins with the endogenous receptors of target neurotransmitters (Sabatini and Tian, 2020). Although powerful, these indirect approaches are limited in their ability to image neurotransmitters only at places where reporters are expressed; therefore, other aspects, such as trafficking inside cells, diffusion, and uptake after exocytosis, cannot be traced. To capture the various phases of neurotransmitters, the combination of which determines real chemical transmission, direct imaging of these small messengers is warranted.

Direct imaging of acetylcholine was performed using the neuromuscular junction of a frog, which is a classical sample used in neuroscience that is highly enriched with acetylcholine (Fu et al., 2017). However, apart from such cases, the direct imaging of endogenous neurotransmitters has rarely been achieved. For example, released dopamine and norepinephrine can be monitored by surface-enhanced Raman spectroscopy outside the cells (Dijkstra et al., 2007). However, imaging them inside the cells is difficult. This is because neurotransmitters are synthesized from abundant molecules. Both dopamine and norepinephrine are generated from, and therefore share most of their chemical groups (and thereby Raman signals) with tyrosine, which exists ubiquitously inside cells as an amino acid. This difficulty may be overcome using a Raman tag in combination with CRS. Thus, alkyne-tagged neurotransmitters have been developed for direct imaging of neurotransmitters (Nuriya et al., 2021; Nakamura et al., 2022). Importantly, these alkyne-tagged neurotransmitters mimic the original untagged neurotransmitters, making them Raman-distinguishable and biologically indistinguishable probes for neurotransmitters. As expected, the alkyne-tagged dopamine exhibited a specific Raman scattering signal in the silent region. However, the millimolar level of detection limits direct imaging of neurons. Further developments in CRS with higher sensitivity, combined with Raman-tagged neurotransmitters, are expected to reveal the dynamics of neurotransmitters and, thereby, the nature of chemical signal transduction.

### 3.3 Water imaging

The brain is a water-rich organ with water accounting for ~80% of its weight. Therefore, metabolic reactions, chemical signal transduction, and other biological phenomena occur in the brains of aquatic environments. Consequently, any changes in water content or its dynamics in the brain are as effective as changes in solutes (e.g., neurotransmitters) in controlling brain function. Consequently, water dynamics have gained considerable attention as key factors in the physiology, pathophysiology, and pharmacology of the brain. Specifically, the “glymphatic system” hypothesis, in which the glia-regulated flow of water inside the brain functions as a lymphatic system by clearing waste molecules that accumulate in the parenchyma, sheds new light on the role of water dynamics in the brain (Iliff et al., 2012). Although controversies exist regarding the detailed mechanisms of the glymphatic system, accumulating evidence supports its importance in pathophysiology in Alzheimer’s and other diseases. Although this concept relies on the dynamics of water, the actual dynamics of water in brain tissue remain poorly characterized. Currently, direct imaging of water dynamics is limited to macroscopic imaging at the whole-brain level using magnetic resonance imaging (Alshuhri et al., 2021). To better understand the nature of water dynamics in the brain and thereby the (patho)physiology of the brain, higher-resolution cellular-level direct imaging is indispensable. The combination of Raman tags and CRS microscopy can resolve this issue.

Previous studies used deuterated water (heavy water, D_2_O) as Raman-tagged water to investigate the dynamics of water in cells and cysts (Potma et al., 2001; Ibata et al., 2011; Yu et al., 2013). These studies have demonstrated that water dynamics can be visualized and characterized at a cellular resolution by imaging D_2_O. Considering the high tissue penetration and three-dimensional spatial resolution of multiphoton microscopy, CRS would be able to image water dynamics inside brain tissues. Therefore, this system was used to monitor the influx of water into acutely prepared murine cortical slices (Shinotsuka et al., 2023). The flow of D_2_O inside living brain tissues was successfully visualized by CRS, together with that of solutes (fluorescent dyes) detected by simultaneous two-photon excitation. This comparison of the molecular movements revealed distinct routes of water and dye flux into the brain. Future studies with higher spatiotemporal resolution, quantification, and *in vivo* imaging, among others, will elucidate water dynamics and thereby complete our knowledge of the regulation of biochemical reactions inside healthy and diseased brain tissues.

### 3.4 Drug imaging

Although different drug modalities have become increasingly popular, more than half of the drugs currently in use are categorized as small-molecule drugs with molecular weights of <500 (Southey and Brunavs, 2023). These drugs are too small for fluorescence tagging, and thus, their pharmacokinetics remain unclear at the cellular and tissue levels, despite their importance in the understanding of drug sites of action. If they were labeled with Raman tags, they could be visualized using CRS.

Several attempts have been made to achieve this objective (Dunnington et al., 2024). For example, alkyne tags were introduced into the antidepressant backbone to create a Raman-tagged analog of a selective serotonin uptake inhibitor and were visualized using surface-enhanced Raman scattering (Tanuma et al., 2020). Importantly, the introduction of an alkynyl group allowed for the specific detection of the compound in brain tissue while preserving the pharmacological properties of the original compound. However, a drawback of the alkyne-tagging strategy is that it requires the synthesis of new compounds for Raman imaging. As noted above, many functional groups can be considered Raman tags, including carbon–deuterium (C—D) bonds. Importantly, many deuterium-substituted compounds have been developed for other purposes and are now available as Raman-tagged drugs. As a proof-of-concept, we employed this strategy to image the widely used general anesthetic propofol in dissociated cultured hippocampal neurons (Oda et al., 2022). Propofol is very small (molecular weight: 178); thus, its subcellular localization could not be analyzed. Deuterium-substituted propofol preserved the original pharmacological properties of propofol while exhibiting specific Raman signals, which were successfully observed in live neurons by SRS, revealing a rather nonspecific plasma membrane distribution of propofol in neurons. Time-lapse imaging was used to monitor the dissociation of drugs from the neurons, allowing for kinetic analyses. With an increased signal-to-noise ratio, this strategy is expected to reveal pharmacokinetics at unprecedented spatial and temporal resolutions in living neurons and brain tissue.

## 4 Discussion and perspectives

CRS can significantly revolutionize neuroscience research by visualizing objects that cannot be visualized using fluorescence imaging. If the neuroscience community knows more about this technique and realizes that it has great potential for realizing imaging analyses of target molecules and/or phenomena that cannot be visualized with conventional techniques, the true power of this technique will be unleashed, contributing unprecedented knowledge to neuroscience.

CRS imaging continues to evolve through the active incorporation of technical advancements into fluorescence imaging. For example, tissue clearing has been combined with CRS to achieve a better performance in deep-tissue volumetric imaging (Li et al., 2019; Shi et al., 2022b). Superresolution CRS imaging has been developed based on various principles (Shi et al., 2022a; Jang et al., 2023; Shou et al., 2023). With such continuous developments in CRS imaging fueled by developments in other fields, the power of CRS imaging continues to expand, providing researchers with further analytical power. However, at the same time, some technical leaps are required to improve the use of CRS in neuroscience. First, the low sensitivity of this technique compared to fluorescence continues to be a limitation of CRS. Several ideas have been introduced for Raman probes and detection systems; however, we have yet to observe minimally invasive and highly sensitive Raman tag imaging regimes that can be applied in various neuroscience studies. The second aspect pertains to the nature of the microscopy system. Although some commercial systems are currently available, most CRS imaging is performed using custom-made microscopes. The flexibility of these custom-made systems is valuable for creating an optimum setup for individual targets; however, it requires skills in optics to build and use such systems. Readily accessible and easy-to-use commercial systems are required for CRS imaging to be widely used in neuroscience research. Finally, the applications of CRS are relatively limited, particularly compared to versatile fluorescent imaging. However, this last point has changed with the introduction of Raman-tag imaging as well as different types of techniques developed in the last 25 years of CRS research. Further attempts at CRS imaging in neuroscience will require technical improvements, the achievements of which will, in turn, expand further applications. The positive feedback between neuroscience applications and basic technical improvements in CRS will contribute to advancements in neuroscience.
